# Cementless calcar-guided short stems: 24-month clinical outcomes and migration analysis

**DOI:** 10.1038/s41598-026-67610-x

**Published:** 2026-08-21

**Authors:** Stefan Budde, Michael Schwarze, Christof Hurschler, Peter Fennema, Henning Windhagen, Jochen Plagge, Thilo Floerkemeier, Gabriela von Lewinski, Yvonne Noll, Alexander Derksen

**Affiliations:** 1 Department of Orthopaedic Surgery, Hannover Medical School, DIAKOVERE Annastift, Anna Von Borries Str. 1-6, 30625 Hannover, Germany; 2 Evangelisches Klinikum Bethel, Universitätsklinikum OWL der Universität Bielefeld, Burgsteig 13, 33617 Bielefeld, Germany; 3 Department for Medical Technology, Hochschule Bremerhaven, An Der Karlstraße 8, 27568 Bremerhaven, Germany; 4 AMR Advanced Medical Research, Männedorf, Switzerland

**Keywords:** Calcar-guided short stem, Total hip arthroplasty, Radiostereometric analysis, Implant migration, Axial subsidence, Anatomy, Diseases, Health care, Medical research

## Abstract

Calcar-guided short stems aim to preserve proximal bone stock and reconstruct individual anatomy, but reduced diaphyseal anchorage raises concerns regarding initial stability. This prospective cohort study evaluated migration of the A2 calcar-guided short stem using high-precision radiostereometric analysis (RSA). Sixty patients (mean age 57.8 years) undergoing primary total hip arthroplasty were enrolled. Marker-based RSA was performed at baseline and at predefined intervals up to 24 months. The primary endpoint was axial subsidence (Ty) at 2 years. Secondary outcomes included three-dimensional migration and clinical scores, including the Harris Hip Score (HHS). Longitudinal migration was analysed using linear mixed models. At 24 months, mean axial subsidence (Ty) was − 0.29 mm (95% CI − 0.53 to − 0.06). Mean total translation (Txyz) was 0.74 mm (95% CI 0.50 to 0.98), and mean retroversion (Ry) was 2.31° (95% CI 1.84° to 2.79°). Migration occurred predominantly in the early postoperative phase, followed by stabilisation. Two stems (3.3%) required revision during follow-up, one due to undersizing with insufficient cortical contact and one after a traumatic fall. The remaining implants demonstrated stable fixation. Mean HHS exceeded 95 at 24 months. The A2 calcar-guided short stem showed early settling followed by stable fixation, with excellent clinical outcomes at 2 years.

## Introduction

Total hip arthroplasty (THA) improves quality of life in patients with irreversible hip joint damage by reducing pain and reliably restoring mobility^1^. Although conventional long-stem prostheses show good success rates, they also pose specific challenges, particularly in revision cases^2^. In response to these challenges, the use of short stems in hip arthroplasty has increased, especially in younger patients with longer life expectancy and the associated higher lifetime risk of revision^2,3^. The advantages of short stems include more conservative preservation of proximal bone stock, which may provide more favorable conditions for future revision procedures^3,4^. In addition, they promote a more physiological load transfer to the proximal femur, potentially resulting in a lower incidence of stress-shielding reactions^3,4^. Furthermore, these implants are considered suitable for minimally invasive approaches due to their shape and reduced length^5–7^. Reflecting these advantages, interest in short stems has risen substantially, and their use in Germany has approximately doubled over the past 10 years, reaching 23.1% of all THA procedures in 2023, according to the German Arthroplasty Registry^8^.

The A2 cementless short stem (ARTIQO GmbH, Lüdinghausen, Germany) is a calcar-guided implant that has been in clinical use since 2016. This implant is designed to emphasize controlled metaphyseal anchorage along the medial femoral calcar, distinguishing it from conventional short stems^6^. Two geometries (Type B and Type G) are available, which are designed to accommodate different femoral anatomies. Scholars have shown that calcar-guided short stems can effectively reconstruct the femoroacetabular offset and maintain leg length, which are crucial for restoring physiological hip function^9^. In particular, a closer reconstruction of the anterior offset may be facilitated by the femoral neck–guided implantation of short stems compared to conventional straight stems^10^. This positioning is assumed to reduce the risk of component impingement while simultaneously improving hip joint function during flexion and internal rotation^10^. Consequently, stem orientation is not dictated by the diaphyseal axis, which enables positioning that is independent of the femoral diaphysis^6^.

The early mechanical behavior of newly introduced femoral stem designs should be evaluated against established performance benchmarks. Radiostereometric analysis (RSA) is particularly well suited for this purpose, as it provides highly accurate assessments of implant migration and has been shown to be predictive of long-term implant survival in as little as 2 years^11,12^. Migration data obtained by RSA provide insight into both primary stability, which is achieved immediately after final intraoperative implant positioning, and secondary stability, which develops over time through osseointegration^12,13^. Adequate primary stability is a prerequisite for successful secondary fixation, and although early postoperative migration reflects mechanical settling, continued or progressive migration beyond this phase has been associated with an increased risk of aseptic loosening^12^.

Early RSA findings from the present cohort at 6 months postoperatively have been previously reported^14^. The present study extends these observations with findings on the migration behavior of the cementless calcar-guided A2 short stem up to 24 months postoperatively. RSA results are summarized descriptively, and clinical outcomes are analyzed longitudinally in the overall cohort. The primary objective of this study was to assess the magnitude and temporal pattern of migration of the A2 short stem over a 24-month follow-up period using RSA. The secondary objectives included the evaluation of clinical outcomes over time in the same cohort. Based on previously published RSA data for cementless short-stem femoral implants, it was hypothesized that subsidence of the A2 short stem would remain within the established migration ranges reported for clinically successful short-stem designs over a 24-month follow-up period. Published RSA results of the Metha short-stem implant (Aesculap AG, Tuttlingen, Germany) serve as an external reference framework for contextual interpretation of migration magnitude^15^. In addition, it was hypothesized that the A2 short stem would demonstrate early stabilization following an initial migration phase, which is consistent with the successful osseointegration of cementless femoral components.

## Materials and methods

### Study design and patient cohort

This investigation was conducted as a prospective single-center cohort study of the migration behavior and clinical performance of the cementless A2 calcar-guided short stem over a follow-up period of 24 months. Between January 2019 and December 2020, 60 consecutive patients undergoing primary THA were enrolled and treated with the cementless A2 short stem.

The present manuscript pertains to the within-cohort migration behavior and clinical course of the A2 short stem assessed by RSA and standardized clinical scores, with Metha serving as an external contextual reference for migration^15^.

The study was approved by the Ethics Committee of Hannover Medical School (No. 7663MPG-LKP Mono, October 2018) and was registered in the German Clinical Trials Register (DRKS00017645). Written informed consent was obtained from all participants.

Surface coating allocation was randomized in the cohort as part of the original study protocol; however, the present analysis was explicitly designed and powered to evaluate the overall migration behavior of the A2 short stem rather than compare coating subgroups. Accordingly, no between-group inference is performed here.

### Primary and secondary RSA endpoints

The primary endpoint of this study was femoral stem migration at 24 months assessed by RSA, with signed axial subsidence (Ty) designated as the primary outcome measure for formal hypothesis testing, in accordance with contemporary RSA standards^16^. Total translation (Txyz), defined as √(Tx^2^ + Ty^2^ + Tz^2^)^14^, and migration along the remaining translational (Tx and Tz) and rotational (Rx, Ry, and Rz) degrees of freedom were analyzed as secondary endpoints.

### Inclusion and exclusion criteria

Patients were eligible for inclusion if they required primary THA for advanced hip arthritis confirmed by plain radiographs or femoral head osteonecrosis (Association Research Circulation Osseous [ARCO] stage III–IV) confirmed by MRI. The additional inclusion criteria were age between 30 and 70 years and normal neurological function. The exclusion criteria were previous bony or soft-tissue surgery of the proximal femur (excluding arthroscopic procedures), active or prior hip joint infection, systemic infection, diagnosed osteoporosis, centrum-collum-diaphyseal (CCD) angles above 145° or below 120°, severe cardiovascular disease (American Society of Anesthesiologists [ASA] class III–IV), known allergy to prosthesis components, neurological disorders affecting motor function, pregnancy or lactation, BMI greater than 30 kg/m², and known alcohol dependence or other relevant substance abuse. Subsequent exclusion criteria included intraoperative fractures of the proximal femur or acetabulum; the intraoperative need to switch to a different implant system; revision surgery due to infection, loosening, or fracture (excluding superficial soft-tissue infections within the first 6 postoperative weeks); withdrawal of informed consent; fewer than four differentiable tantalum markers on postoperative RSA images; and an RSA condition number exceeding 120.

### Implant characteristics

The cementless A2 calcar-guided short stem is manufactured from forged titanium alloy (Ti6Al4V) and features an open-pore titanium plasma spray (TPS) coating, which is optionally combined with an additional calcium phosphate (CaP) coating (Bonit, DOT, Rostock, Germany). Two stem geometries are available—Type B and Type G—which differ in curvature to facilitate anatomical reconstruction of individual femoral morphologies^6^. Type B is available in sizes 2–11, and Type G is available in sizes 1–9. All procedures were performed using ceramic-on-polyethylene bearings. The acetabular component consisted of either the ANA.NOVA Alpha cup or the ANA.NOVA Hybrid cup (ARTIQO GmbH, Lüdinghausen, Germany). Only ceramic femoral heads with diameters of 28–32 mm were used.

### Surgical technique and postoperative management

Preoperative digital planning was performed using OrthoView™ version 7.1.5 (Materialise, Leuven, Belgium) to determine implant size and alignment based on individual patient anatomy. All procedures were carried out by three experienced surgeons, who used the minimally invasive anterolateral modified Watson–Jones approach to minimize soft-tissue trauma. Femoral preparation and stem implantation were performed according to the manufacturer’s standardized surgical technique. Following definitive implant placement, 6 to 10 biocompatible tantalum markers (1.0 mm in diameter; Tilly Medical Products AB, Lund, Sweden) were implanted into the proximal femur in the regions of the greater and lesser trochanter to serve as fiducial markers for RSA.

Postoperative rehabilitation followed a standardized protocol with full weight-bearing, as tolerated under physiotherapeutic supervision. The first postoperative mobilization occurred after baseline RSA imaging within the first 24 h after surgery.

Perioperative antibiotic prophylaxis consisted of cefuroxime 1.5 g i.v.; postoperative thromboprophylaxis was performed with certoparin 3000 IU. Both followed institutional standard protocols.

### RSA analysis

Migration behavior of the femoral stem was evaluated using model-based RSA. The coordinate system was defined by the calibration cage, with the x-axis positive in the medial direction, the y-axis positive in the cranial (proximal) direction, and the z-axis positive in the anterior direction; rotations (Rx, Ry, and Rz) were defined about these axes according to the right-hand rule^15,17,18^. RSA images were analyzed using the software RSAcore version 4.11 (Leiden University Medical Center, Leiden, Netherlands). Double examinations were performed in 15 patients in accordance with ISO 16087:2013, which yielded a translational precision of 0.27 mm. RSA image acquisition and analysis were performed by trained technical personnel according to a standardized protocol. To minimize assessment bias, RSA analysis was performed without using implant allocation labels as analytic input.

### Timing of assessments

Baseline RSA images were obtained within the first 24 h after surgery, prior to full weight-bearing. Subsequent RSA examinations were performed on postoperative day 7, at 6 weeks, and at 3, 6, 12, and 24 months.

### Clinical and radiographic evaluation

Clinical assessments were performed preoperatively and at 3, 6, 12, and 24 months postoperatively. They included the Harris Hip Score (HHS)^19^, Hip Disability and Osteoarthritis Outcome Score (HOOS)^20^, HOOS-Joint Replacement Score (HOOS-JR)^21^, UCLA activity score^22^, RAND 36 version 1.0^23^, Physical and Mental Component Summary Scores^24^, Forgotten Joint Score (FJS-12)^25^, and visual analog scale (VAS; 0–10) for pain.

Standardized anteroposterior pelvic and Lauenstein radiographs were obtained postoperatively and at 3, 12, and 24 months for safety monitoring.

### Sample size calculation

The protocol’s sample size planning was based on a one-sided noninferiority framework versus Metha as a historical reference. Specifically, the protocol described using a historical Metha RSA dataset from the study center with a mean subsidence of 0.86 mm (SD 0.97 mm)^15^, while assuming a mean subsidence of 0.40 mm for the A2 short stem in the absence of prior data. Conservative assumptions were specified (alpha = 0.025, power = 0.90), which yielded a minimum required sample size of 48 patients for a one-sided comparison. Anticipating a 20% dropout rate, 60 patients were targeted for enrollment.

### Statistical analysis

Baseline demographic characteristics and clinical outcome scores were summarized using descriptive statistics (mean ± SD and frequencies). Changes in clinical outcome scores between the preoperative and 24-month assessments were evaluated using the Wilcoxon signed-rank test. Based on the 2024 International RSA Guidelines, the statistical analysis prioritized signed axial subsidence (Ty) over vector magnitudes (Txyz) to reduce bias in unsigned summary metrics, which increases with measurement error^16^. Consequently, for RSA endpoints, formal hypothesis testing was restricted to the Ty axis.

Longitudinal migration data were analyzed using a linear mixed model (LMM), with time entered as a categorical fixed effect. To account for serial correlation in repeated measures, a first-order autoregressive (AR1) residual covariance structure was utilized, which provided a significantly better fit than a standard random-intercept model (AIC > 10, *p* < 0.001). The model-estimated marginal mean for Ty at 24 months was formally compared to the historical reference value using a postestimation linear contrast.

Steady-state reliability was assessed for the period between 3 months and 2 years using intraclass correlation coefficients (ICCs), with higher ICC values indicating greater signal-to-noise and stable positioning. The longitudinal RSA model was specified to estimate migration behavior conditional on osseointegration; therefore, stems revised for early mechanical failure were excluded from the LMM but retained in clinical survival analyses. Two-sided *p* values < 0.05 were deemed statistically significant. Analyses were performed with Stata version 15.1 (Stata Corp, College Station, TX, USA).

## Results

### Study population and demographics

**Fig. 1:**
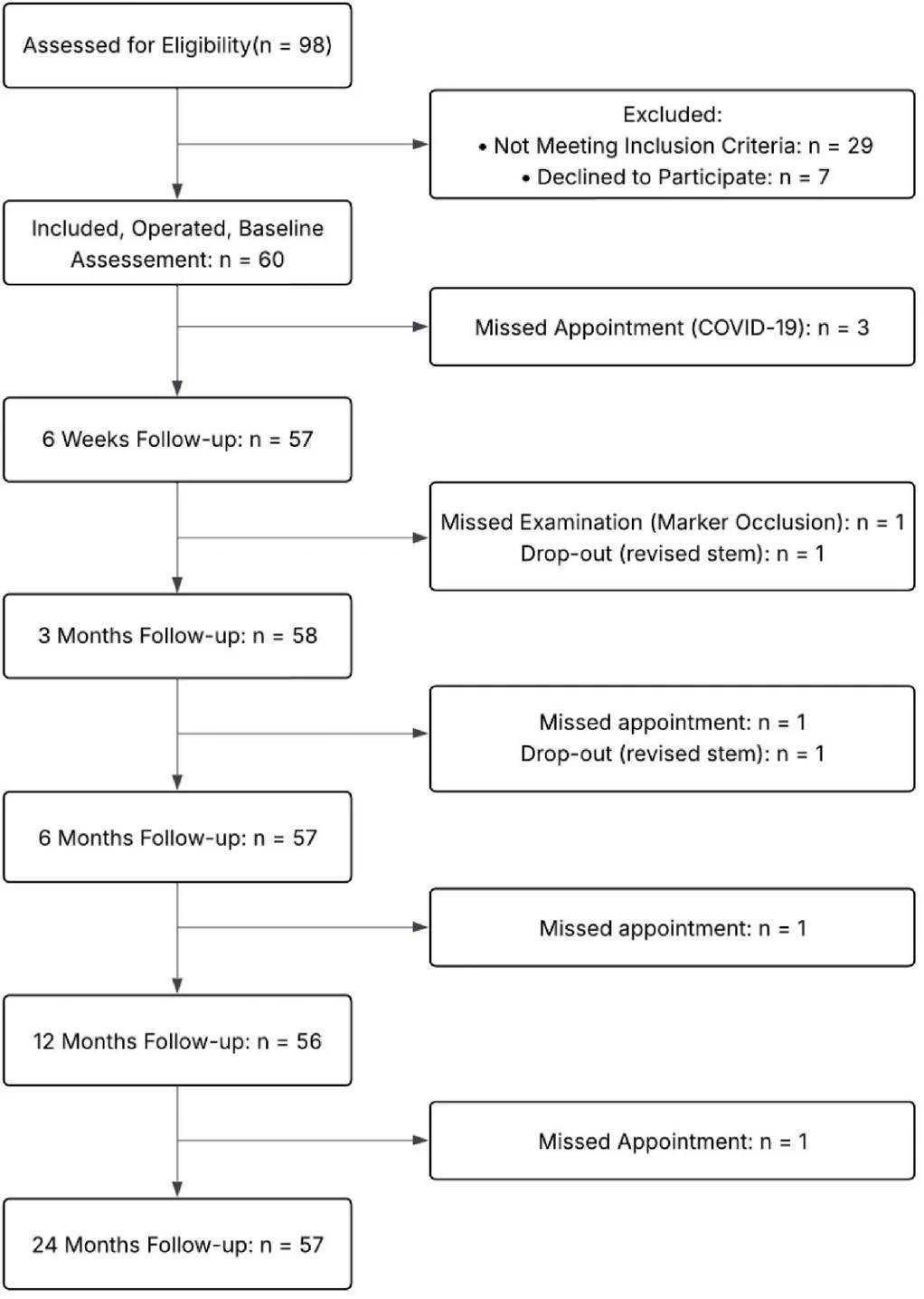
Study flow of the participants during the study period.

The study flow is depicted in Fig. 1. The study cohort comprised 60 patients undergoing primary THA; their baseline characteristics are summarized in Table 1.

**Table 1:** Baseline demographics and implant characteristics.

Characteristic	Total cohort (*n* = 60)
Age (years), mean ± SD (range)	57.8 ± 8.5 (30–70)
Sex, n (%)	
Female	38 (63.3)
Male	22 (36.7)
BMI (kg/m²), mean ± SD (range)	24.9 ± 2.8 (19.3–29.7)
Weight (kg), mean ± SD (range)	74.7 ± 12.3 (50–98)
Diagnosis, n (%)	
Primary osteoarthritis	50 (83.3)
Dysplasia	9 (15.0)
Other	1 (1.7)
Implant coating, n (%)	
Titanium plasma spray	30 (50.0)
Calcium phosphate	30 (50.0)
Stem variant (shape), n (%)	
Type B	47 (78.3)
Type G	13 (21.7)

### Follow-up and exclusions

Due to state-imposed containment measures during the COVID-19 pandemic, three patients were unable to attend the 6-week follow-up visit. Over the 24-month study period, individual RSA frames were excluded for four patients due to the following technical factors: occlusion of tantalum beads (*n* = 1), high condition number > 120 (*n* = 1), incomplete image capture (*n* = 1), and incorrect capture of the contralateral hip (*n* = 1). Reimaging was declined in these instances. Additionally, sporadic missed visits occurred at 6 months (*n* = 1), 12 months (*n* = 2), and 24 months (*n* = 1) due to patient refusal unrelated to the implant.

### Clinical outcomes

Clinical outcome scores improved substantially from baseline to 24 months, with statistically significant changes for all measures except the RAND-36 MCS (Table 2). The mean HHS improved from 49.8 preoperatively to 95.4 at 2 years; the HOOS-JR score improved from 46.0 to 92.6. High functional performance was reflected in the UCLA activity score (mean 6.9 at 24 months) and the FJS-12, which reached a mean of 75.1 at the final follow-up. All HOOS subscales achieved “very good” results by 24 months, with particular gains noted in the “pain” (95.1) and “activities of daily living” (95.0) domains.

**Table 2:** Clinical outcomes (Mean ± SD).

Outcome measure	Preoperative	3 months	6 months	12 months	24 months	*p*, baseline vs. 24 mo
HHS	49.8 ± 13.0	87.8 ± 11.8	92.8 ± 7.8	95.1 ± 6.3	95.4 ± 6.4	< 0.001
UCLA activity	5.2 ± 1.6	5.9 ± 1.4	7.0 ± 1.3	6.8 ± 1.1	6.9 ± 1.1	< 0.001
FJS-12	–	52.8 ± 27.3	66.6 ± 26.2	78.7 ± 24.1	75.1 ± 25.0	–
HOOS-JR	46.0 ± 15.7	84.9 ± 12.5	91.1 ± 11.0	93.9 ± 9.8	92.6 ± 9.9	< 0.001
HOOS subscales						
Symptoms	41.2 ± 16.8	85.5 ± 11.6	89.7 ± 11.2	91.6 ± 12.2	92.9 ± 9.4	< 0.001
Pain	41.4 ± 13.8	89.6 ± 12.1	93.3 ± 9.6	95.9 ± 8.1	95.1 ± 8.2	< 0.001
ADL	43.9 ± 13.8	85.9 ± 12.7	92.2 ± 10.0	94.4 ± 9.1	95.0 ± 7.3	< 0.001
Sports/rec	19.1 ± 14.8	68.5 ± 23.4	81.3 ± 18.2	86.9 ± 15.5	88.8 ± 13.8	< 0.001
QoL	21.6 ± 14.4	64.7 ± 19.8	76.5 ± 16.7	83.0 ± 15.4	83.2 ± 15.5	< 0.001
RAND-36						
PCS	28.2 ± 6.7	46.6 ± 8.2	51.6 ± 7.4	52.7 ± 7.0	53.0 ± 6.3	< 0.001
MCS	50.0 ± 11.6	55.2 ± 6.1	53.5 ± 5.4	53.1 ± 5.3	51.4 ± 8.7	0.784

Values presented as mean ± standard deviation. HHS = Harris Hip Score; FJS-12 = Forgotten Joint Score; HOOS = Hip Disability and Osteoarthritis Outcome Score; ADL = activities of daily living; QoL = quality of life; PCS = Physical Component Score; MCS = Mental Component Score.

### Stem survival and complications

Two stems (3.3%) required revision within the first 6 months, resulting in a 24-month stem survival rate of 96.7%. One revision was performed at 6 weeks for symptomatic aseptic loosening associated with undersizing and lack of distal cortical contact. The second revision was performed 3 months postoperatively after a traumatic fall on ice, which precipitated severe symptoms and a Trendelenburg gait. Both cases were classified as clinical failures and were excluded from the longitudinal RSA migration analysis, which was intended to characterize the migration behavior of the osseointegrated cohort.

Two dislocations occurred during follow-up; one was managed with closed reduction and one required liner exchange. Neither dislocation required stem revision. One intraoperative adverse event involved significant blood loss requiring transfusion. In all complication cases, the patients recovered without permanent functional impairment at subsequent follow-up.

### Subsidence (Ty)

**Fig. 2:**
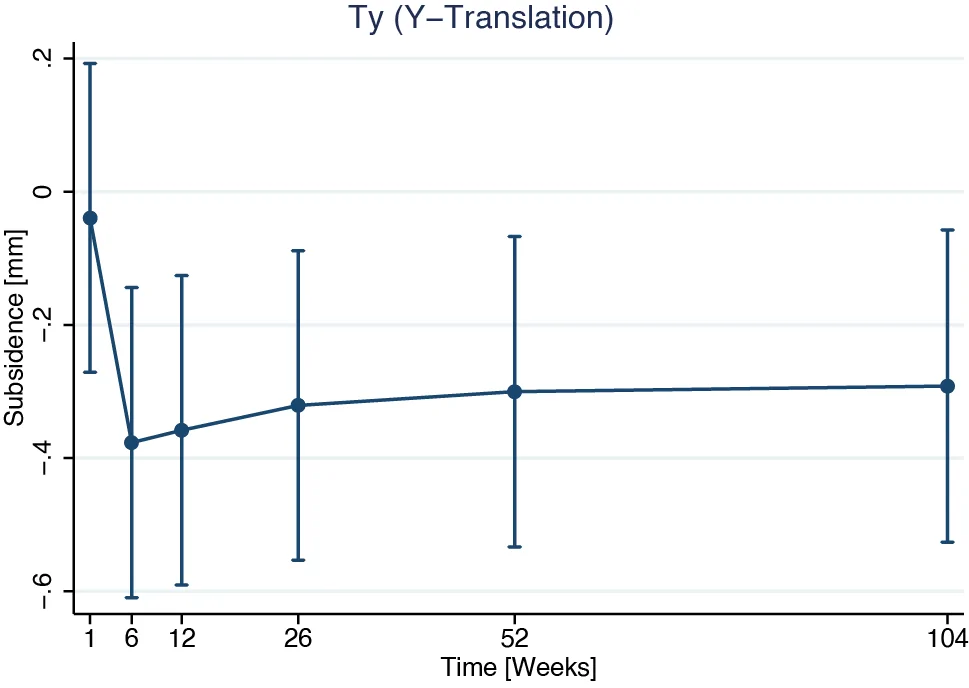
Longitudinal Y-translation migration of the A2 femoral stem measured by RSA (model-based mean ± 95% CI).

Longitudinal analysis showed greater migration between baseline and 6 weeks than between 6 weeks and 24 months, with minimal further change thereafter. The model-estimated mean axial subsidence (Ty) at 24 months was − 0.29 mm (95% confidence interval [CI]: −0.53 to − 0.06) (Fig. 2; Table 3), which was below the prespecified historical reference value of 0.86 mm (*p* < 0.001).

**Table 3:** Stem Migration presented as model-based mean (95% CI).

Motion parameter	Timepoint	A2 stem (*n* = 58)
Y-translation (subsidence)	1 week	− 0.04 (− 0.27 to 0.19)
*(proximal +/distal* −*)*	6 weeks	− 0.38 (− 0.61 to − 0.14)
	3 months	− 0.36 (− 0.59 to − 0.13)
	1 year	− 0.32 (− 0.55 to − 0.09)
	2 years	− 0.29 (− 0.53 to − 0.06)
X-translation	1 week	0.04 (− 0.04 to 0.12)
*(medial +/lateral* −*)*	6 weeks	0.07 (− 0.01 to 0.15)
	3 months	0.13 (0.05 to 0.20)
	1 year	0.16 (0.08 to 0.24)
	2 years	0.13 (0.05 to 0.21)
Z-translation	1 week	− 0.06 (− 0.18 to 0.05)
*(anterior +/posterior* −*)*	6 weeks	− 0.11 (− 0.22 to 0.01)
	3 months	− 0.18 (− 0.30 to − 0.07)
	1 year	− 0.20 (− 0.31 to − 0.08)
	2 years	− 0.17 (− 0.28 to − 0.05)
Y-rotation (version)	1 week	0.72 (0.26 to 1.18)
*(retro +/ante* −*)*	6 weeks	1.17 (0.70 to 1.64)
	3 months	2.04 (1.58 to 2.51)
	1 year	2.38 (1.92 to 2.85)
	2 years	2.31 (1.84 to 2.79)
X-rotation (tilt)	1 week	0.18 (− 0.02 to 0.38)
*(anterior +/posterior* −*)*	6 weeks	0.33 (0.13 to 0.54)
	3 months	0.38 (0.18 to 0.58)
	1 year	0.53 (0.33 to 0.73)
	2 years	0.47 (0.27 to 0.68)
Z-rotation (varus/valgus)	1 week	− 0.25 (− 0.44 to − 0.06)
*(valgus +/varus* −*)*	6 weeks	− 0.32 (− 0.51 to − 0.13)
	3 months	− 0.33 (− 0.52 to − 0.15)
	1 year	− 0.28 (− 0.47 to − 0.09)
	2 years	− 0.28 (− 0.47 to − 0.09)
Total translation	2 years	0.74 (0.50 to 0.98)
Max total point motion	2 years	2.34 (1.99 to 2.69)

One biological outlier was identified in the surviving cohort. This implant exhibited high-magnitude initial subsidence (− 7.7 mm at 6 weeks), which was attributed to undersizing; however, continuous RSA monitoring showed secondary stability, with only 0.12 mm of additional migration between 3 months and 2 years.

### Rotation and total translation

**Fig. 3:**
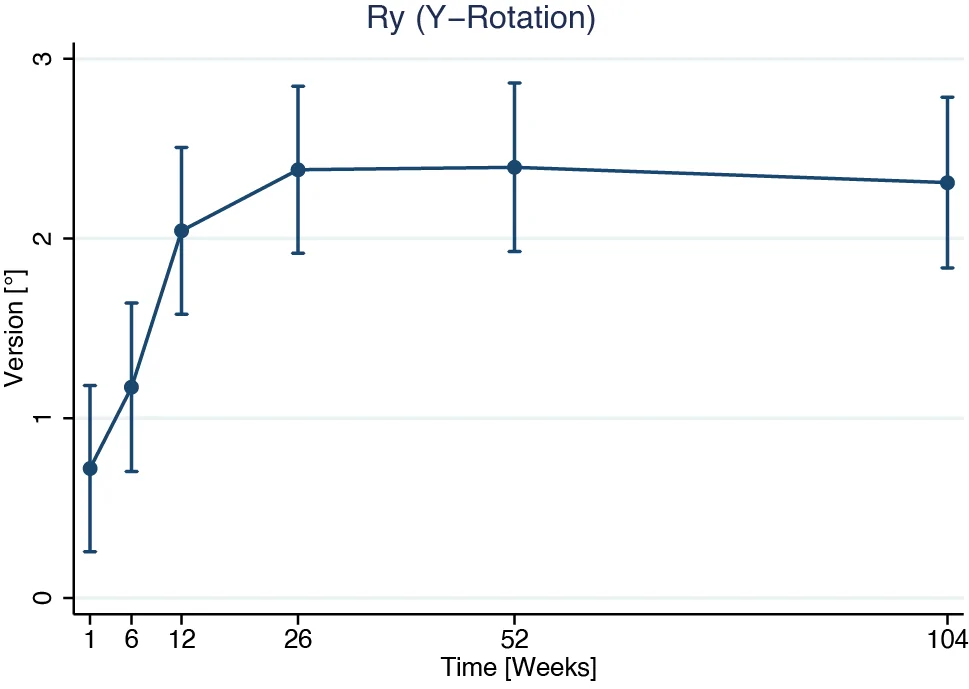
Longitudinal Y-rotation of the A2 femoral stem measured by RSA (model-based mean ± 95% CI).

Mean retroversion (Ry) at 24 months was 2.31° (95% CI: 1.84° to 2.79°) (Fig. 3), which was similar to the 1-year value of 2.38°, with no further increase observed between 6 months and 24 months. Mean total translation (Txyz) at 24 months was 0.74 mm (95% CI: 0.50 to 0.98), which exceeded the signed subsidence (Ty).

Steady-state variance components and ICCs were calculated for the interval between 12 weeks and 104 weeks (Table 4). The ICC for axial subsidence (Ty) was 0.77. For total translation (Txyz), the ICC was 0.81. Regarding the rotational components, the ICC values were 0.69 for Ry and 0.00 for Rz. The estimated between-subject variance for Rz was < 0.001.

**Table 4:** Variance components and intraclass correlation coefficients (ICCs) for the steady-state period (3 months to 2 years).

Motion parameter	Between-subject variance	Residual (intra-subject) variance	Total variance	ICC
Translations (mm)				
X-translation (Tx)	0.063	0.023	0.086	0.73
Y-translation (Ty)	0.141	0.042	0.183	0.77
Z-translation (Tz)	0.194	0.057	0.251	0.77
Total translation (Txyz)	0.210	0.048	0.258	0.81
Rotations (deg)				
X-rotation (Rx)	0.533	0.193	0.726	0.73
Y-rotation (Ry)	2.307	1.017	3.324	0.69
Z-rotation (Rz)	0.000	0.556	0.556	0.00*
Max total point motion	1.223	0.378	1.601	0.76

For the Z-rotation, the between-subject variance was negligible (< 0.0001), indicating uniform stability across the cohort rather than poor reliability.

## Discussion

The primary aim of this study was to evaluate the migration behavior and long-term fixation of the A2 short stem using high-precision RSA.

The results demonstrate a migration pattern consistent with rapid and durable osseointegration. The primary endpoint, subsidence (Ty) at 24 months, was − 0.29 mm (95% CI: −0.53 to − 0.06), which indicates limited vertical migration and a high degree of stability. This finding aligns with the favorable clinical outcomes, with the HHS improving to > 95 points and the FJS exceeding the ROC-derived patient acceptable symptom state threshold of 66.7^26^.

The steady-state variance analysis provided insight into migration behavior after the early postoperative period. From 12 weeks to 24 months, the ICC values for Ty (0.77) and Ry (0.69) show that most of the variability during this interval reflected stable between-subject differences rather than continued within-subject change, suggesting that individual stems tended to maintain their relative settled positions over time.

In contrast, the transverse translation components showed low between-subject variance and higher residual variance, which constrains the interpretability of vector-based migration metrics and highlights the limited precision of these axes in RSA. Accordingly, although Txyz captures overall displacement, its magnitude should be interpreted with caution in short-stem applications where transverse precision is inherently lower.

Taken together, these findings support the use of signed axial subsidence (Ty) and rotational migration (Ry) as the most informative descriptors of fixation behavior over time.

These migration results compare favorably with historical standards for short-stem fixation. The A2 stem demonstrated a mean Ty of − 0.29 mm at 24 months, indicating less distal migration than the historical Metha reference used for sample-size planning (*p* < 0.001). When compared in its specific design class, the A2 aligns closely with established peers, such as the Optimys and Nanos stems, showing lower migration than older metaphyseal-anchoring designs (Table 5).

**Table 5:** Comparison of 24-month migration patterns by stem design class.

Stem class	Stem design	Study	*N*	Subsidence (Ty)	Retroversion (Ry)	Total migration (Txyz)
Calcar guided	A2 stem	Current study	58	− 0.29 mm	2.31°	0.74 mm
	Optimys	De Waard et al.^17^	33	− 0.21 mm	1.15°	NR
	Nanos	Budde et al.^27^	14	− 0.22 mm	0.83°	0.46 mm
Curved triple tapered	Fitmore	Acklin et al.^28^	34	− 0.39 mm	1.09°	NR
Tapered wedge	Tri-Lock	Jørgensen et al.^29^	48	− 0.38 mm	1.68°	2.01 mm
Metaphyseal	Metha	Floerkemeier et al.^18^	39	− 0.72 mm	3.50°	1.00 mm

The subsidence for Optimys is presented as a median in the source text. NR = not reported.

Rotational behavior followed a distinct pattern of early migration followed by stabilization. The A2 stem exhibited a mean retroversion (Ry) of 2.31° at 2 years. This value places it in an intermediate position in the short-stem spectrum: It rotates more than the Nanos (0.83°)^27^ and Optimys (1.15°)^17^ but substantially less than the metaphyseal-anchoring Metha stem (3.50°)^18^. The combined axial and rotational migration pattern raises the hypothesis that fixation may involve both calcar seating and rotational engagement in the metaphysis. Ry increased predominantly during the early postoperative period, and the steady-state ICC for Ry (0.69) indicates that most of the variance from 3 months to 24 months reflected stable between-subject differences rather than progressive migration (Table 4). As shown by De Waard et al. in the Optimys cohort, even stems with high initial migration can achieve secure secondary stability without compromising clinical outcomes^17^.

The total translation vector (Txyz = 0.74 mm) exceeded the signed axial subsidence (Ty = − 0.29 mm), which might at first glance suggest clinically relevant multiaxial motion. However, this difference reflects both the mathematical properties of vector magnitudes and the mechanical behavior of calcar-guided stems. Because Txyz is calculated as √(Tx² + Ty² + Tz²), all translational components contribute positively, such that small motions in the transverse axes, together with unavoidable measurement variability in each axis, inflate the vector magnitude even in the absence of true progressive migration. When the true translational components are small, as in a well-stabilized stem, this measurement variability can become the dominant contributor to the vector magnitude.

In the present cohort, Ty showed substantial between-subject variance and high intraclass correlation, indicating that axial subsidence captured patient-specific settling and secondary stability. In contrast, the transverse axes displayed distinct mechanical patterns. Anteroposterior translation (Tz) exhibited greater between-subject variability than Ty, consistent with patient-specific differences in anterior–posterior toggling during metaphyseal seating, whereas medio-lateral translation (Tx) remained small. Varus–valgus rotation (Rz) demonstrated individually stable trajectories after the early postoperative period, with each stem settling into a patient-specific equilibrium position, reflecting constrained mechanical behavior about the medial calcar rather than measurement noise.

Consequently, although Txyz provides a summary of overall displacement, its magnitude is disproportionately influenced by the combination of small transverse motions and the unsigned nature of the vector metric, particularly once stabilization has occurred. In this context, signed subsidence (Ty) and rotational behavior around the stem axis (Ry) provide the most direct and interpretable measures of biological fixation and secondary stability, while the remaining components describe complementary mechanical modes of interface engagement. A similar discrepancy between vector magnitude and axial subsidence has been reported for other well-fixed stems, such as the Tri-Lock design, where Txyz exceeded axial migration despite stable fixation^29^.

This study has several limitations. First, it was conducted at a single center with a relatively limited sample size. Second, although the original protocol was randomized by surface coating (TPS versus CaP), the early coating-specific RSA findings from this cohort have been reported separately, and the present manuscript was intentionally scoped at the cohort level. Third, RSA studies vary in protocol details—notably the timing of the baseline reference relative to first post-operative loading, marker handling, and outcome definitions—and measurement precision is partly determined by stem geometry itself; direct quantitative comparison of migration values across cohorts and historical controls should therefore be interpreted with caution. Fourth, RSA quantifies in vivo migration but does not by itself identify the underlying fixation mechanism; elucidating the mechanistic basis of the observed rotational pattern would require a separately designed mechanistic study. Finally, although the 24-month follow-up is appropriate for early RSA-based assessment of fixation behavior, longer-term clinical and radiographic follow-up will be required to confirm durability of fixation.

In conclusion, the A2 calcar-guided short stem demonstrated excellent early clinical performance and rapid stabilization, with a mean vertical subsidence of − 0.29 mm at 2 years that aligns with the established Nanos and Optimys designs. Ry increased predominantly during the early postoperative period and stabilized thereafter; the absence of relevant progression after 3–6 months suggests early rotational settling followed by secondary stability rather than progressive rotational migration. Clinically, the findings support favorable short-term fixation and clinical performance of the A2 short stem in appropriately selected patients, while emphasising the importance of correct sizing and stable cortical engagement. Continued clinical follow-up and registry surveillance remain important to confirm durability of fixation beyond the 24-month RSA window.

## Data Availability

The datasets used and/or analysed during the current study available from the corresponding author on reasonable request.

## References

[1] Learmonth, I. D., Young, C. & Rorabeck, C. The operation of the century: Total hip replacement. *Lancet* **370**, 1508–1519 (2007). 17964352 10.1016/S0140-6736(07)60457-7

[2] Gruner, A. & Heller, K. D. Patient selection for shorter femoral stems. *Orthopedics* **38**, 27–32 (2015). 10.3928/01477447-20150215-53 25826629

[3] Migliorini, F. et al. Short stems for total hip replacement among middle-aged patients. *Int. Orthop.* **44**, 847–855 (2020). 32193611 10.1007/s00264-020-04516-x

[4] Loppini, M. & Grappiolo, G. Uncemented short stems in primary total hip arthroplasty: The state of the art. *EFORT Open Rev.* **3**, 149–159 (2018). 29951251 10.1302/2058-5241.3.170052 PMC5994625

[5] de Waard, S. et al. Short-term success of proximal bone stock preservation in short hip stems: A systematic review of the literature. *EFORT Open. Rev.* **6**, 1040–1051 (2021). 34909223 10.1302/2058-5241.6.210030 PMC8631238

[6] Kutzner, K. P. Calcar-guided short-stem total hip arthroplasty: Will it be the future standard? Review and perspectives. *World J. Orthop.* **12**, 534–547 (2021). 34485100 10.5312/wjo.v12.i8.534 PMC8384612

[7] Castelli, C. C. & Rizzi, L. Short stems in total hip replacement: Current status and future. *Hip Int.* **24**(Suppl 10), S25–28 (2014). 24970038 10.5301/hipint.5000169

[8] Grimberg, A. et al. *Annual Report 2024* (The German Arthroplasty Registry (EPRD), 2025).

[9] Amenabar, T., Marimuthu, K., Hawdon, G., Gildone, A. & McMahon, S. Total hip arthroplasty using a short-stem prosthesis: Restoration of hip anatomy. *J. Orthop. Surg.* **23**, 90–94 (2015). 25920653 10.1177/230949901502300121

[10] Ezechieli, M. et al. A neck-preserving short stem better reconstructs the centre of rotation than straight stems: A computed tomography-based cadaver study. *Arch. Orthop. Trauma Surg.* **142**, 1669–1680 (2022). 34231044 10.1007/s00402-021-03957-2

[11] Bottner, F. et al. Radiostereometric analysis: The hip. *HSS J.* **1**, 94–99 (2005). 18751815 10.1007/s11420-005-0114-2 PMC2504129

[12] de Vries, L. M. et al. The predictive value of radiostereometric analysis for stem survival in total hip arthroplasty. A systematic review. *Hip Int.* **24**, 215–222 (2014). 24474413 10.5301/hipint.5000102

[13] Kim, J. T. & Yoo, J. J. Implant design in cementless hip arthroplasty. *Hip Pelvis* **28**, 65–75 (2016). 27536647 10.5371/hp.2016.28.2.65 PMC4972888

[14] Budde, S. et al. Very early migration of a calcar-guided short stem: A randomized study of early mobilization and the influence of a calcium phosphate coating with 60 patients. *Sci. Rep.* **14**, 3837 (2024). 38360840 10.1038/s41598-023-50829-3 PMC10869691

[15] Schwarze, M. et al. No effect of conventional vs. minimally invasive surgical approach on clinical outcome and migration of a short stem total hip prosthesis at 2-year follow-up: A randomized controlled study. *Clin. Biomech.* **51**, 105–112 (2018). 10.1016/j.clinbiomech.2017.12.004 29287171

[16] Kaptein, B. L. et al. Guideline for RSA and CT-RSA implant migration measurements: An update of standardizations and recommendations. *Acta Orthop.* **95**, 256–267 (2024). 38819193 10.2340/17453674.2024.40709 PMC11141406

[17] de Waard, S. et al. The migration pattern and initial stability of the Optimys short stem in total hip arthroplasty: A prospective 2-year follow-up study of 33 patients with RSA. *Hip Int.* **31**, 507–515 (2021). 31971010 10.1177/1120700020901844

[18] Floerkemeier, T. et al. Greater early migration of a short-stem total hip arthroplasty is not associated with an increased risk of osseointegration failure: 5th-year results from a prospective RSA study with 39 patients, a follow-up study. *Acta Orthop.* **91**, 266–271 (2020). 32106733 10.1080/17453674.2020.1732749 PMC8023937

[19] Harris, W. H. Traumatic arthritis of the hip after dislocation and acetabular fractures: treatment by mold arthroplasty. An end-result study using a new method of result evaluation. *J. Bone Jt. Surg.* **51-A**, 737–755 (1969). 5783851

[20] Blasimann, A., Dauphinee, S. W. & Staal, J. B. Translation, cross-cultural adaptation, and psychometric properties of the German version of the hip disability and osteoarthritis outcome score. *J. Orthop. Sports Phys. Ther.* **44**, 989–997 (2014). 25394689 10.2519/jospt.2014.4994

[21] Lyman, S. et al. Validation of the HOOS, JR: A short-form hip replacement survey. *Clin. Orthop. Relat. Res.* **474**, 1472–1482 (2016). 26926772 10.1007/s11999-016-4718-2 PMC4868170

[22] Zahiri, C. A., Schmalzried, T. P., Szuszczewicz, E. S. & Amstutz, H. C. Assessing activity in joint replacement patients. *J. Arthroplasty* **13**, 890–895 (1998). 9880181 10.1016/s0883-5403(98)90195-4

[23] Hays, R. D., Sherbourne, C. D. & Mazel, R. M. The RAND 36-item health survey 1.0. *Health Econ.* **2**, 217–227 (1993). 8275167 10.1002/hec.4730020305

[24] Farivar, S. S., Cunningham, W. E. & Hays, R. D. Correlated physical and mental health summary scores for the SF-36 and SF-12 Health Survey, V.I. *Health Qual. Life Outcomes*. **5**, 54 (2007). 17825096 10.1186/1477-7525-5-54 PMC2065865

[25] Behrend, H., Giesinger, K., Giesinger, J. M. & Kuster, M. S. The forgotten joint as the ultimate goal in joint arthroplasty: Validation of a new patient-reported outcome measure. *J. Arthroplasty*. **27**, 430–436e431 (2012). 22000572 10.1016/j.arth.2011.06.035

[26] Singh, V. et al. The Forgotten Joint Score patient-acceptable symptom state following primary total hip arthroplasty. *Bone Jt. Open.* **3**, 307–313 (2022). 35387474 10.1302/2633-1462.34.BJO-2022-0010.R1 PMC9044089

[27] Budde, S. et al. Analysis of migration of the Nanos(R) short-stem hip implant within two years after surgery. *Int. Orthop.* **40**, 1607–1614 (2016). 26404094 10.1007/s00264-015-2999-9

[28] Acklin, Y. P., Jenni, R., Bereiter, H., Thalmann, C. & Stoffel, K. Prospective clinical and radiostereometric analysis of the Fitmore short-stem total hip arthroplasty. *Arch. Orthop. Trauma Surg.* **136**, 277–284 (2016). 26739137 10.1007/s00402-015-2401-9

[29] Jørgensen, P. B., Homilius, M., Koppens, D., Hansen, T. B. & Stilling, M. Similar femoral stem fixation but less metaphyseal loss of bone mineral density with a taper-wedge design and diaphyseal bone preservation with a long and round-tapered design: A 5-year randomized RSA and DXA study of 50 patients. *Acta Orthop.* **96**, 656–663 (2025). 40891927 10.2340/17453674.2025.43907 PMC12404101

